# Prediction of Mental Health in Medical Workers During COVID-19 Based on Machine Learning

**DOI:** 10.3389/fpubh.2021.697850

**Published:** 2021-09-07

**Authors:** Xiaofeng Wang, Hu Li, Chuanyong Sun, Xiumin Zhang, Tan Wang, Chenyu Dong, Dongyang Guo

**Affiliations:** ^1^Northeast Asian Research Center, Jilin University, Changchun, China; ^2^Kuancheng Health Commission, Changchun, China; ^3^Department of Social Medicine and Health Management, School of Public Health, Jilin University, Changchun, China

**Keywords:** COVID-19, mental health, prediction, machine learning, artificial intelligence, neural network, public health

## Abstract

Mental health prediction is one of the most essential parts of reducing the probability of serious mental illness. Meanwhile, mental health prediction can provide a theoretical basis for public health department to work out psychological intervention plans for medical workers. The purpose of this paper is to predict mental health of medical workers based on machine learning by 32 factors. We collected the 32 factors of 5,108 Chinese medical workers through questionnaire survey, and the results of Self-reporting Inventory was applied to characterize mental health. In this study, we propose a novel prediction model based on optimization algorithm and neural network, which can select and rank the most important factors that affect mental health of medical workers. Besides, we use stepwise logistic regression, binary bat algorithm, hybrid improved dragonfly algorithm and the proposed prediction model to predict mental health of medical workers. The results show that the prediction accuracy of the proposed model is 92.55%, which is better than the existing algorithms. This method can be used to predict mental health of global medical worker. In addition, the method proposed in this paper can also play a role in the appropriate work plan for medical worker.

## Introduction

Although the definition of mental health is not uniform in academic circles, the research significance of mental health is self-evident. Mental health has been widely used in psychology (1), sociology (2), psychiatry (3), pedagogy (4, 5), genetics (6), and other fields.

Currently, some representative scales are usually used to measure mental health, such as Self-reporting Inventory (SCL-90) (7), Minnesota Multiphasic Personality Inventory (MMPI) (8), Self-Rating Anxiety Scale (SAS) (9), Self-Rating Depression Scale (SDS) (10), Eysenck Personality Questionnaire (EPQ) (11), the Sixteen Personality Factor Questionnaire (16PF) (12). Above scales are widely used internationally because they are guided by various psychological theories and can transform abstract mental health concepts into observable specific indicators. However, some shortcomings are not considered in the scales mentioned above. First, the different emphasis of scale measurement leads to the differences in the evaluation criteria because many factors need to be considered in the measurement of mental status. Second, the existing way of answering the scales is self-evaluation, which inevitably makes the respondent hold something back. Third, a lot of time is spent in obtaining the results of the scale for judging mental status in emergency situations. Although the diagnosis and intervention of mental symptoms are significant, prevention is even more important. Therefore, using existing information to predict mental health is of great significance.

Mental health prediction is conductive to detecting mental disorders in advance, reducing the incidence of serious mental illnesses, and facilitating the health system to provide people with targeted health care services (13). In particular, the mental health of medical workers is seriously threatened by the global spread of COVID-19. These workers are prone to anxiety and depression (14). United Nations Secretary-General António Guterres indicated in “Message on COVID-19 and the demand for action of mental health” (15) that various mental health services must be shifted to the community and must be included in the all-people medical plan. Based on a survey conducted by WHO, COVID-19 pandemic has caused the disruption of major mental health services in 93% of countries worldwide (16). However, there are urgent demand for mental health services in many countries. In addition, the delta variants have appeared in at least 98 countries and regions, and continue to mutate and evolve. Almost all new cases of CIVID-19 are the delta variants (17), and the delta variants are becoming the main epidemic strain in many countries. The delta variant pandemic is likely to further exacerbate the fears of the public and medical workers. Therefore, predicting the potential psychological symptoms of medical workers contributes to the mental health of medical personnel, and helps maintain the high efficiency of global medical institutions.

The existing mental health prediction methods are divided into statistical model methods and artificial intelligence algorithms.

Among the statistical model methods that are used for mental health prediction, structural equation models are widely used (18–20). Moving average methods are also commonly used in health prediction. Autoregressive Integrated Moving Average model (ARIMA) (21–23) and Exponential Smoothing (ES) (24, 25) are the representative methods of the moving average model. Negative binomial model (NBM) (26, 27) and fractional polynomials (28) also provide new mentalities for predicting health. However, the statistical analysis of the data can be achieved by the above methods, but the inherent relationship between the characteristic variables and the prediction results cannot be identified by the above methods. Therefore, the accuracy of mental health prediction based on statistical models is low.

For the purpose of improving the accuracy of mental health prediction, machine learning technology has been used in the mental health prediction research since the 1980s. Basavappa et al. (29) proposed a depth-first search method according to reverse search strategy in 1996, which is used to diagnose depression or dementia. Basavappa et al. developed an expert system based on the subjects' behavior, cognition, symptoms of emotion, and neuropsychological assessment results. Gil. and Manuel (30) come up with a system according to Artificial Neural Network (ANN) and Support Vector Machine (SVM) in 2009, which is used to diagnose Parkinson's disease. The system improves the accuracy of diagnosis and reduces the cost of diagnosis. Seixas et al. (31) come up with a model called Bayesian Network (BN) in 2014, which is used to diagnose dementia and Alzheimer disease. The experimental results show that compared with most other well-known classifiers, the BN decision model has better performance. Dabek and Caban (32) proposed a neural network model in 2015, which is used to assess the possibility of suffering from psychological illnesses.

However, three problems are not solved in the algorithms mentioned above. First, statistical models are difficult to tackle the impact of random interference factors on mental health because of their limitations. Therefore, statistical models cannot reflect the high uncertainty of mental health and the non-linear relationship between feature variables and prediction results, which leads to their low prediction accuracy. Second, existing machine learning methods that are used for mental health prediction only focus on prediction accuracy without considering the impact of feature variables on the importance of mental health. Therefore, the influence weight cannot be determined according to the degree to which feature variables have effects on the prediction results. As a result, the above algorithms cannot provide a theoretical basis for the health department to work out psychological intervention plans for medical workers. Third, a large number of irrelevant or redundant features are usually included in the datasets that are used for mental health prediction. Statistical model methods merely choose significant features rather than important features, which cannot eliminate the irrelevant and redundant features in dataset that influence prediction results. Consequently, the mental health prediction methods based on statistical model not only have low prediction accuracy, but also waste computing time.

To deal with the above problems, the article proposes a novel mental health prediction algorithm called the Improved Global Chaos Bat Back Propagation Neural Network (IGCBA-BPNN). The purpose of this article is to monitor the mental health of medical workers in time to reduce the incidence of mental illness of medical workers, and to rationalize the distribution of global public health resources. Therefore, IGCBA-BPNN is applied to the mental health prediction of Chinese medical workers. The experimental results show that, compared with the existing mental health prediction methods, IGCBA-BPNN not only improves the accuracy of mental health prediction, but also selects the fewest feature variables.

The contribution of this paper is the proposal of a new mental health prediction algorithm. The proposed algorithm can predict more effectively the mental health of medical workers during COVID-19, and at the same time provides a theoretical basis for global public health departments to work out psychological intervention plans.

The remaining content of this article is arranged as followed: in section Materials and Methods, we introduced the data and methods of this research. In section Results, the effectiveness of proposed algorithm is evaluated. At last, the discussion and conclusion are illustrated in detail.

## Materials and Methods

### Data Preparation

Using dataset from the “Mental Health Status of Medical Workers During COVID-19” survey conducted in Changchun, Jilin Province, China from June 1, 2020 to June 7, 2020, this paper predicts the mental health of Chinese medical workers during COVID-19. The subjects of above survey are medical workers who participated in epidemic prevention and control. According to the population status and the characteristics of geographical distribution, we selected 150 grass-roots medical units from 220 grass-roots medical units in the Changchun city and then randomly selects 35 medical workers in each grass-roots medical unit. The questionnaire is conducted online and 5,260 questionnaires were obtained in this survey. Based on research need, 152 unqualified samples were eliminated and the final sample size is 5,108. There are 32 variables in the questionnaire. In the process of designing the questionnaire, we collected as much as possible the basic information of the subjects and the variables information that may affect mental status of medical workers during COVID-19. Studies have shown that the measurable factors affecting mental status mainly include the five respects of demography (33, 34), family (35, 36), employment (37, 38), lifestyle (39, 40), and work/living environment related to COVID-19 (41, 42). Based on the results of the existing literature and the actual situation of medical workers during COVID-19, 32 factors were decided.

The description of variables is presented in Table 1. The data and its description are published on GitHub (https://github.com/Hu-Li/mental-health-dataset).

**Table 1:** The description of variables.

**Factors**	**Variables**	**Variable type**
Demography	Gender	Unordered
	Age	Numeric
	Place of residence	Unordered
	Town or country	Unordered
	Education	Ordered
	Marital status	Unordered
	Chronic disease	Unordered
Family	The only child	Unordered
	Have minor children or not	Unordered
	Whether the minor child is an only child	Unordered
	Primary caregiver for children	Unordered
	Primary caregiver for elderly parents	Unordered
	Annual family income	Numeric
	Current job is supported by family	Unordered
Employment	Occupation	Unordered
	Post	Unordered
	Working years	Numeric
	Work units nature	Unordered
	Title	Unordered
	Employment type	Unordered
	Monthly income	Numeric
	Changes in work intensity	Unordered
	Working hours per week	Numeric
	Satisfaction level with the protective measures	Unordered
	Psychological training	Unordered
Lifestyle	Usual sleep time	Numeric
	Resting place	Unordered
	Frequency of exercise	Unordered
Work/living environment	Have COVID-19 patients or not in the workplace	Unordered
related to	In close contact with COVID-19 patients in the workplace	Unordered
COVID-19	Have COVID-19 patients or not in the living place	Unordered
	The work unit is a designated treatment point or not	Unordered

This study had been reviewed and approved by the Ethics Committee of the School of Public Health, Jilin University. This study does not involve questions about the identity of the respondents. An informed consent page was provided on the first page of the questionnaire for confirmation. All participants voluntarily joined this study with informed consent.

### Feature Selection

#### Bat Algorithm

The Bat Algorithm (BA) (43) proposed by Yang is widely used in many fields because of its simplicity, fast convergence speed and few parameters. The bat algorithm has been used by many scholars for feature selection (44, 45). The excellent performance of the bat algorithm has also been verified in comparison with other most well-known algorithms such as genetic algorithm (GA) and particle swarm optimization (PSO) (46). Bat algorithm uses echolocation principles to simulate the predation process of bats. Bat algorithm is also an effective search method, and it is used to search for the global optimal solution. Original bat algorithm has three ideal hypotheses so as to simulate the predation behavior of bats:

First, bats use echolocation to perceive the distance between themselves and the target, and they can effectively distinguish targets and obstacles. Second, the *i*th bat flies randomly at a speed *v*_*i*_ in the space position *x*_*i*_, and searches for targets with frequency *f*_*i*_, wavelength λ and loudness *A*_*i*_. Bats adjust the rate of emission of pulse *r*(*r* ∈ [0,1]) according to the distance between themselves and prey. Third, loudness changes from maximum *A*_max_ to minimum *A*_min_.

Based on the above three ideal hypotheses, in the search space, the calculating equations of the frequency, velocity and position of bats as follows:

(1) $$\begin{array}{l} f_{i}=f_{min}+\left(f_{max}-f_{min}\right)\times \beta \end{array}$$

(2) $$\begin{array}{l} v_{i}^{t+1}=v_{i}^{t}+\left(x_{i}^{t}-x_{*}\right)\times f \end{array}$$

(3) $$\begin{array}{l} x_{i}^{t+1}=x_{i}^{t}+v_{i}^{t+1} \end{array}$$

where *f*_*i*_ is the pulse frequency of the *i*th bat, and *f*_min_ and *f*_max_ are the minimum and maximum value of the pulse frequency, respectively, β is a random number within [0,1], $v_{i}^{t+1}$ is the flight speed of the *i*th bat at the *t* + 1th iteration, $v_{i}^{t}$ is the flight speed of the *i*th bat at the *t*th iteration, $x_{i}^{t}$ is the position where the *i*th bat stays at the *t*th iteration, $x_{i}^{t+1}$ is the position where the *i*th bat stays at the *t* + 1th iteration, x_*_ is the optimal position of the bat in the current population.

In the process of searching for prey, the initial ultrasonic loudness of bats is large, but the emission rate is low. This helps bats search for prey in the entire space. When a bat finds prey, the loudness of volume that the bat emits is gradually reduced, and the rate of emission of pulse is gradually increased. Through the above adjustments, bats can more accurately determine the location of prey. The rate of emission of pulse and the loudness of volume that the bat emits are calculated as follows:

(4) $$\begin{array}{l} r_{i}^{t+1}=r_{i}^{0}\left[1-\exp\left(-\gamma \times t\right)\right] \end{array}$$

(5) $$\begin{array}{l} A_{i}^{t+1}=\alpha \times A_{i}^{t} \end{array}$$

where $r_{i}^{t+1}$ is the pulse emission rate of the *i*th bat at the *t* + 1th iteration, $r_{i}^{0}$ is the maximum of pulse emission rate of the *i*th bat, γ(γ > 0) is the enhancement coefficient of the pulse frequency, $A_{i}^{t+1}$ and $A_{i}^{t}$ are the loudness of volume that the *i*th bat emits at the *t* + 1th iteration and at the *t*th iteration, respectively, α(α ∈ [0,1]) is the attenuation coefficient of the pulse loudness.

However, the bat algorithm is easy to fall into the local optimum, and the prediction accuracy of bat algorithm is low. The population initialization of the bat algorithm is randomly generated and does not have the ability to cover the entire solution space, which greatly affects the performance of the bat algorithm.

#### Improved Global Chaos Bat Algorithm

In order to overcome the shortcomings of the bat algorithm, Global Chaos Bat Algorithm (GCBA) (47) is introduced to eliminate redundant features and irrelevant features in the dataset. As a heuristic optimization algorithm, GCBA is used for feature selection. At first, in the initial stage, the chaotic map method is introduced to ensure the bat population traverse the entire solution space as much as possible. The chaotic map method also conducive to enriching the population diversity. Then, a fitness function based on accuracy and feature subset length is proposed to calculate the score of the feature subset after each update. Finally, GCBA selects the feature subset with the highest score from all feature subsets through the score calculated, which eliminate irrelevant features and redundant features from all feature variables.

To further improve the performance of GCBA, Improved Global Chaos Bat Algorithm (IGCBA) with higher accuracy and better performance is proposed, in which a nonlinear function based on the number of iterations is designed to balance IGCBA's exploitation and exploration capabilities. In the early stage of IGCBA, the algorithm is inclined toward the exploration capability. Global information is fully utilized to enable IGCBA to traverse the entire solution space as much as possible. In the later stage of IGCBA, the algorithm is inclined toward exploitation capability. Partial information is fully utilized to enable IGCBA to obtain the better solution through further exploitation.

Currently, the logistic method is widely used as a chaotic map method. The initial population generated by this method is diverse and can traverse the entire solution space. Therefore, in this paper, the initialization of the population is finished by using an improved logistic mapping method, and its mathematical model (48) is:

(6) $$\begin{array}{l} y_{i}^{d+1}=|1-2\times {\left(y_{i}^{d}\right)}^{2}| \end{array}$$

where $y_{i}^{d}\left(i=1,2,\cdots N,d=1,2,\cdots D\right)\left(y_{i}^{d}\in \left[0,1\right]\right)$ is the chaotic variable, *N* is the amount of bat population, and *D* is the dimension of initial population. Then, the position $x_{i}^{d}$ of the bat individual in the solution space is obtained by inverse mapping of $y_{i}^{d}$. The calculating equation of $x_{i}^{d}$ is:

(7) $$\begin{array}{l} x_{i}^{d}=l_{i}+\left(u_{i}-l_{i}\right)y_{i}^{d} \end{array}$$

where *l*_*i*_ and *u*_*i*_ are the minimum and maximum value of the variable range, respectively.

The local optimum position of the bat and the global optimum position of the population are recorded when the position of each bat is updated. The position of the *i*th bat at the *t* + 1th iteration can be calculated as follows:

(8) $$\begin{array}{l} x_{i}^{t+1}=x_{i}^{t}+v_{i}^{t+1}C_{1}r_{1}\left(P_{i}-x_{i}^{t}\right)+C_{2}r_{2}\left(P_{g}-x_{i}^{t}\right) \end{array}$$

where *P*_*i*_ is the local optimal position of the *i*th bat, *P*_*g*_ is the global optimal position of the bat population, *r*_1_ and *r*_2_ are two random numbers within [0,1].

*C*_1_ is the control coefficient that balances the global exploration capability of IGCBA, represents the degree to which the historical optimal position of a bat individual has effect on the current state of the bat. The larger the *C*_1_ is, the more the algorithm focuses on exploitation capability. *C*_2_ is the control coefficient that balances the local exploitation capability of IGCBA, represents the degree to which the historical optimal position of the bat population has effect on the current state of the bat. The larger the *C*_2_ is, the more the algorithm focuses on exploration capability.

In the preliminary stage of algorithm, it is necessary to traverse the entire solution space as much as possible to ensure that the algorithm does not converge prematurely. Therefore, in the early stage of the algorithm, *C*_2_ should be as large as possible and *C*_1_ as small as possible; in the later stage of the algorithm, *C*_1_ should be as large as possible and *C*_2_ as small as possible. In this way, the algorithm can get better performance. According to the above analysis, the calculating equation of *C*_1_ and *C*_2_ as follows:

(9) $$C_{1}=\{\begin{array}{c} e^{-(\frac{T}{2}-t)/10}+0.1,\;0\leq t<40\\ 0.0095\times t-0.0980,\;40\leq t<70\\ 4.8+\frac{20}{\mathrm{loge}(t+70)},\;70\leq t<100 \end{array}$$

(10) $$C_{2}=\{\begin{array}{c} 0.9-e^{-(\frac{T}{2}-t)/10},\;0\leq t<40\\ 0.0095\times t+0.9120,\;40\leq t<70\\ \frac{-20}{\mathrm{loge}(t+70)}-3.8,\;70\leq t<100 \end{array}$$

where *t* represents the current iteration times, *T* represents the maximum iteration times.

When initializing the bat population, we use a matrix of size *N* × *D*. *N* is the number of bat population, *D* is the number of features. In this paper, a transfer equation is used to perform discrete binary operations on the bat's position. The transfer equation is:

(11) $$\begin{array}{l} S\left(x_{i}^{d}\left(t\right)\right)=\frac{1}{1+e^{-x_{i}^{d}\left(t\right)}} \end{array}$$

where $x_{i}^{d}\left(t\right)$ is the position of the *i*th bat individual in the *d*th dimension at the *t*th iteration.

The updating equation of position of the bat individual is:

(12) $$x_{i}^{d}\left(t\right)=\{\begin{array}{c} 0,\;rand<S(x_{i}^{d}(t))\\ 1,\;rand\geq S(x_{i}^{d}(t))\; \end{array}$$

where *rand* is a random number within [0,1].

When the *i*th bat's position in the *d*th dimension at the *t*th iteration is 0, this bat will not be selected. When the *i*th bat's position in the *d*th dimension at the *t*th iteration is 1, this bat will be selected.

### Back Propagation Neural Network

Back Propagation Neural Network (BPNN) is particularly suited for solving the non-linear problems (49), so it is widely used in the field of health prediction (50). In the process of back propagation of prediction errors, the connection weights and bias are constantly adjusted. Finally, the output predicted by BPNN is constantly close to the expected output.

Before using BPNN for prediction, the network needs to be trained. Through training, the network will have associative memory and predictive capabilities. The main steps of the BPNN training process are:

Step 1: Initialize the network. Based on the input and output sequence (*X, Y*), the number of the input layer nodes *s* and the output layer nodes *m* can be determined. The number of hidden layers and the number of the hidden layer nodes *l* are given by experience. The connection weight *w*_*hj*_(*h* = 1, 2, ⋯*s*; *j* = 1, 2, ⋯*l*) between the input and the hidden layer, the connection weight *w*_*jk*_(*j* = 1, 2, ⋯*l*; *k* = 1, 2, ⋯ , *m*) between hidden and the output layer, the hidden layer bias value *a*_*j*_ and the output layer bias value *b*_*k*_ are initialized. Given the learning rate η, the activation function *g*(*x*). In order to solve non-linear problems, the activation function usually uses the Sigmoid function, which is defined as follows:

(13) $$\begin{array}{l} g\left(x\right)=\frac{1}{1+e^{-x}} \end{array}$$

Step 2: The output of the hidden layer. The output *H*_*j*_ of the hidden layer is calculated based on the input vector *X*, ω_*hj*_ and *a*_*j*_.

(14) $$\begin{array}{l} H_{j}=g\left(\sum_{h=1}^{n}\omega_{hj}x_{h}+a_{j}\right) \end{array}$$

Step 3: The output of the output layer. The prediction output *O*_*k*_ of BPNN is calculated based on *H*_*j*_, ω_*jk*_ and *b*_*k*_.

(15) $$\begin{array}{l} O_{k}=\sum_{j=1}^{l}H_{j}\omega_{jk}+b_{k} \end{array}$$

Step 4: Calculate prediction error. The prediction error of *p*th simple *E*_*p*_ is calculated based on prediction output of *p*th simple *O*_*pk*_ and expected output of *p*th simple *Y*_*pk*_.

(16) $$\begin{array}{l} E_{p}=\frac{1}{2}\sum_{k=1}^{m}{\left(Y_{pk}-O_{pk}\right)}^{2} \end{array}$$

Step 5: Calculate the reverse transmission value. The reverse transmission value of output layer δ_*k*_, and the reverse transmission value of hidden layer δ_*j*_ are calculated as follows:

(17) $$\begin{array}{l} \delta_{k}=O_{pk}\left(1-O_{pk}\right)\left(Y_{pk}-O_{pk}\right) \end{array}$$

(18) $$\begin{array}{l} \delta_{j}=H_{j}\left(1-H_{j}\right)\sum_{k=1}^{m}\delta_{k}\omega_{jk} \end{array}$$

Step 6: Update the weight. η is the learning rate, and the weight ω_*hj*_ and ω_*jk*_ are updated as follows:

(19) $$\begin{array}{l} \omega_{hj}=\omega_{hj}+\eta \delta_{j}x_{h} \end{array}$$

(20) $$\begin{array}{l} \omega_{jk}=\omega_{jk}+\eta \delta_{k}H_{j} \end{array}$$

Step 7: Update the bias value. The bias value *a*_*j*_ and *b*_*k*_ are updated based on δ_*j*_ and δ_*k*_.

(21) $$\begin{array}{l} a_{j}=a_{j}+\eta \delta_{j} \end{array}$$

(22) $$\begin{array}{l} b_{k}=b_{k}+\eta \delta_{k} \end{array}$$

Step 7: Determine whether the algorithm iteration is over, if not, return to step 2.

### Improved Global Chaos Bat Back Propagation Neural Network

**Figure 1:**
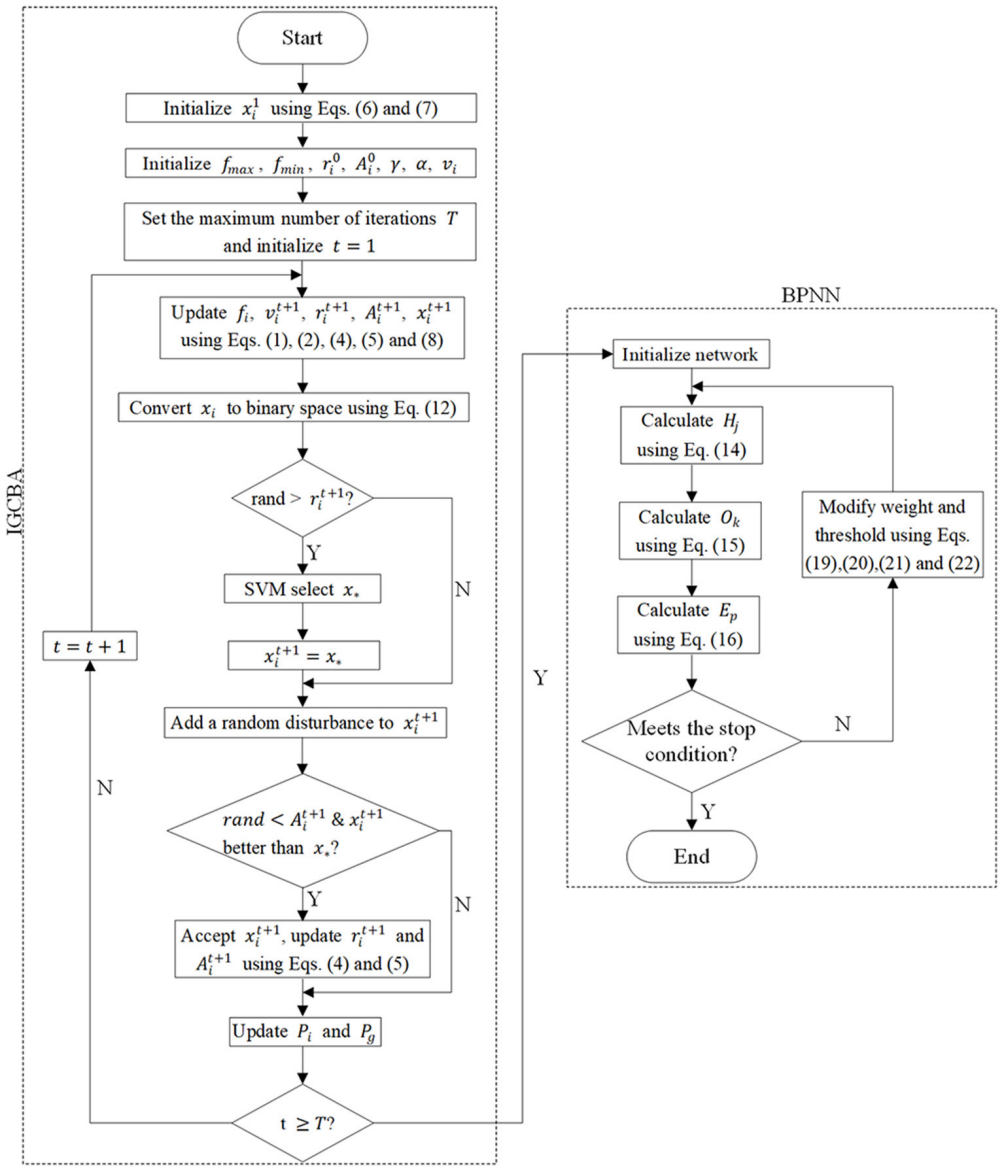
The flowchart of IGCBA-BPNN.

Figure 1 illustrates the process of IGCBA-BPNN. First, initialize all variables. Second, IGCBA is used for feature selection to select a feature subset that can represent as much information as possible of the original features and as few numbers as possible. Existing research has proved that compared with other classifiers, SVM has higher classification accuracy (51) and better stability (52). Therefore, SVM is used to judge the quality of the feature subset selected by IGCBA. Third, the features selected by IGCBA are used as the input of the BPNN to reduce the model complexity of BPNN.

## Results

### Parameter Settings

Table 2 shows the parameter settings of feature selection algorithms. In binary bat algorithm (BBA) (46), GCBA, IGCBA, *A* is the loudness of volume that the bat emits and is set to 1.5, *r* is the rate of emission of pulse and is set to 0.5, *f*_max_ is the maximum value of the pulse frequency and is set to 1, and *f*_min_ is the minimum value of the pulse frequency and is set to 0. In GCBA, *C*_1_ is the control coefficient, represents the degree to which the historical optimal position of a bat individual has effect on the current state of the bat. *C*_1_ is set to 1.49618. *C*_2_ is the control coefficient, represents the degree to which the historical optimal position of the bat population has effect on the current state of the bat. *C*_2_ is set to 1.49618. In hybrid improved dragonfly algorithm (HIDA) (53), *s* and *a* are the separation weight and the alignment weight, respectively, and they are both set to 0.1. *c* is the cohesion weight and is set to 0.7. *f* and *e* are the food factor and the enemy factor, respectively, and they are both set to 1. *w* is the inertia weight and is set to 0.9. In information gain binary butterfly optimization algorithm (IG-bBOA) (54), *N* represents the number of butterflies and is set to 10, *p* is the transition probability and is set to 0.8, *a* is the power exponent and is set to 0.1, *C* is the sensory modality and is set to 0.01-0.25. α, β, and δ are set to 0.99, 0.001, and 0.009, respectively. In hyper learning binary dragonfly algorithm (HLBDA) (55), the parameters of *s*, *a*, *c*, *f*, *e*, and *w* are consistent with HIDA. The *pl* is the personal learning rate and is set to 0.4, and *gl* is the global learning rate and is set to 0.7.

**Table 2:** The parameter settings of feature selection algorithms.

**Algorithm**	**Parameter**	**Meaning**	**Value**	**References**
BBA	*A*	Loudness	1.5	(36)
	*r*	The rate of emission of pulse	0.5	
	*f*_max_	The maximum value of frequency	1	
	*f*_min_	The minimum value of frequency	0	
HIDA	*s*	Separation weight	0.1	(38)
	*a*	Alignment weight	0.1	
	*c*	Cohesion weight	0.7	
	*f*	Food factor	1	
	*e*	Enemy factor	1	
	*w*	inertia weight	0.9	
GCBA	*A*	Loudness	1.5	(37)
	*r*	The rate of emission of pulse	0.5	
	*f*_max_	The maximum value of frequency	1	
	*f*_min_	The minimum value of frequency	0	
	*C*_1_	The control coefficient	1.49618	
	*C*_2_	The control coefficient	1.49618	
IGCBA	*A*	Loudness	1.5	–
	*r*	The rate of emission of pulse	0.5	
	*f*_max_	The maximum value of frequency	1	
	*f*_min_	The minimum value of frequency	0	
IG-bBOA	*N*	Number of butterflies	10	(54)
	*p*	Transition probability	0.8	
	*a*	Power exponent	0.1	
	*C*	Sensory modality	0.01-0.25	
	α	Increasing classification accuracy	0.99	
	β	Reducing the number of features	0.001	
	δ	Increasing the mean of mutual information	0.009	
HLBDA	*s*	Separation weight	0.1	(55)
	*a*	Alignment weight	0.1	
	*c*	Cohesion weight	0.7	
	*f*	Food factor	1	
	*e*	Enemy factor	1	
	*w*	inertia weight	0.9	
	*pl*	Personal learning rate	0.4	
	*gl*	Personal learning rate	0.7	

After combining BPNN with SR, BBA, HIDA, GCBA, IGCBA, IG-bBOA, and HLBDA, the relevant parameters are set in Tables 3, 4. *q* is the number of the hidden layer and is set to 1. *p* is the training goal and is set to 1,000. *g* is the training goal and is set to 1e-4. η is the learning rate and is set to 0.08. *l* is the number of the hidden layer nodes, and as a matter of experience, it is often set to half of the number of input layer nodes. The number of hidden layer nodes of SR-BPNN-4, BBA-BPNN-4, BBA-BPNN-8, HIDA-BPNN-4, HIDA-BPNN-16, GCBA-BPNN-4, GCBA-BPNN-16 and IGCBA-BPNN-4, IG-bBOA-BPNN-4, IG-bBOA-BPNN-10, HLBDA-BPNN-4, and HLBDA-BPNN-14 is set to 4, 4, 8, 4, 16, 4, 9, 4, 4, 10, 4, and 14, respectively.

**Table 3:** Common parameter settings in BPNN.

**Parameter**	**Meaning**	**Value**
*q*	Hidden layer	1
*p*	Training times	1,000
*g*	Training goal	1e-4
η	Learning rate	0.08

**Table 4:** The number of hidden layer nodes in BPNN.

**Algorithm**	**Value**
SR-BPNN-4	4
BBA-BPNN-4	4
BBA-BPNN-8	8
HIDA-BPNN-4	4
HIDA-BPNN-16	16
GCBA-BPNN-4	4
GCBA-BPNN-9	9
IGCBA-BPNN-4	4
IG-bBOA-BPNN-4	4
IG-bBOA-BPNN-10	10
HLBDA-BPNN-4	4
HLBDA-BPNN-14	14

### Experiment Results

We make experiments to compare the IGCBA algorithm with stepwise regression (SR) (56), BBA, HIDA, GCBA, IG-bBOA, and HLBDA methods on the survey dataset in this section. At the same time, we also perform experiments to compare the IGCBA-BPNN algorithm with SR-BPNN, BBA-BPNN, HIDA-BPNN, GCBA-BPNN, IG-bBOA-BPNN and HLBDA methods on the survey dataset. Given that BPNN, K-Nearest Neigbour (KNN) (57) and decision tree (DT) (58) are important methods for classification, we also add the comparison results of BPNN with KNN and DT. Table 5 shows the experimental results.

**Table 5:** Comparison of prediction accuracy of different algorithms.

**Algorithm**	**Number of features**	**The prediction accuracy**
SR	10	87.45%
SR-BPNN-4		88.43%
BBA	15	88.43%
BBA-BPNN-4		88.62%
BBA-BPNN-8		89.02%
HIDA	32	88.63%
HIDA-BPNN-4		90.78%
HIDA-BPNN-16		91.18%
GCBA	18	88.49%
GCBA-BPNN-4		90.20%
GCBA-BPNN-9		90.98%
IG-bBOA	21	88.49%
IG-bBOA-BPNN-4		90.00%
IG-bBOA-BPNN-10		90.39%
HLBDA	29	88.66%
HLBDA-BPNN-4		90.39%
HLBDA-BPNN-14		90.98%
IGCBA	9	88.51%
IGCBA-BPNN-4		92.55%
IGCBA-KNN		87.52%
IGCBA-DT		79.56%

Compared with SR, BBA, HIDA and GCBA, HIDA and HLBDA have the highest prediction accuracy followed by IGCBA. However, the number of features finally found by IGCBA is 23 and 20 fewer than HIDA and HLBDA, respectively. Besides, the number of features selected by IGCBA is also less than other methods. By comparing the performance of the feature selection algorithms, it can be proved that IGCBA can reduce the irrelevant and redundant features in the original features as much as possible without reducing the prediction accuracy of the classifier.

The prediction accuracy of SR-BPNN-4 is 0.98% higher than that of SR. The prediction accuracy of BBA-BPNN-4 and BBA-BPNN-8 is 0.19 and 0.59% higher than that of BBA, respectively. The prediction accuracy of HIDA-BPNN-4 and HIDA-BPNN-16 is 2.15 and 2.55% higher than that of HIDA, respectively. The prediction accuracy of GCBA-BPNN-4 and GCBA-BPNN-9 is 1.71 and 2.49% higher than that of GCBA, respectively. The prediction accuracy of IG-bBOA-BPNN-4 and IG-bBOA-BPNN-10 is 1.51 and 1.90% higher than that of IG-bBOA, respectively. The prediction accuracy of HLBDA-BPNN-4 and HLBDA-BPNN-14 is 1.73 and 2.32% higher than that of HLBDA. The prediction accuracy of IGCBA-BPNN-4 is 4.04% higher than IGCBA. The above experimental results prove that compared with the feature selection algorithms, the feature selection algorithms combined with BPNN can improve the prediction accuracy.

The prediction accuracy of SR-BPNN-4, BBA-BPNN-4, BBA-BPNN-8, HIDA-BPNN-4, HIDA-BPNN-16, GCBA-BPNN-4, GCBA-BPNN-9, IG-bBOA-BPNN-4, IG-bBOA-BPNN-10, HLBDA-BPNN-4, and HLBDA-BPNN-14 is 88.43, 88.62, 89.02, 90.78, 91.18, 90.20, 90.98, 90.00, 90.39, 90.39, and 90.98%, respectively. The prediction accuracy of IGCBA-BPNN-4 is 92.55%, which is 4.12, 3.93, 3.53, 1.77, 1.37, 2.35, 1.57, 2.55, 2.16, 2.16, and 1.57% higher than that of SR-BPNN-4, BBA-BPNN-4, BBA-BPNN-8, HIDA-BPNN-4, HIDA-BPNN-16, GCBA-BPNN-4, GCBA-BPNN-9, IG-bBOA-BPNN-4, IG-bBOA-BPNN-10, HLBDA-BPNN-4, and HLBDA-BPNN-14. The experimental results of combining each feature selection algorithm with BPNN prove that IGCBA-BPNN-4's performance is better than other algorithms. At the same time, the prediction accuracy of IGCBA-KNN and IGCBA-DT is 87.52 and 79.56%, respectively. The prediction accuracy of IGCBA-BPNN-4 is 5.03 and 12.99% higher than that of IGCBA-KNN and IGCBA-DT, respectively. It can be proved that BPNN is better than KNN and DT for classification on survey dataset. Therefore, IGCBA-BPNN-4 model has good applicability in predicting the mental health of medical workers in public health events.

**Figure 2:**
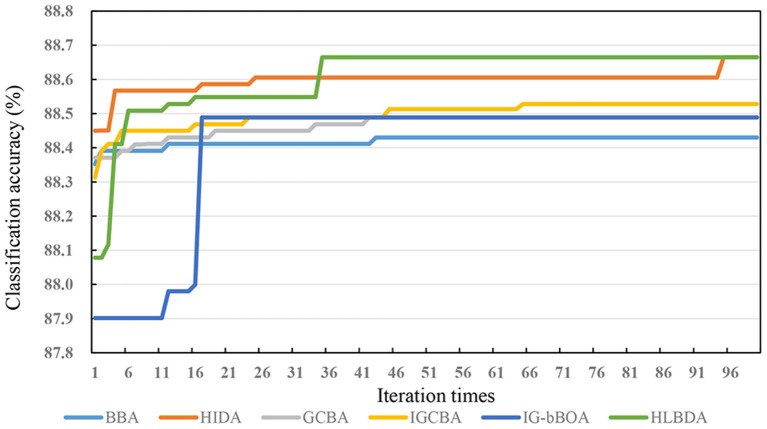
Convergence curves of the six algorithms on the survey dataset.

For the purpose of better verifying the superior convergence performance of the IGCBA algorithm on the test dataset, Figure 2 shows the convergence performance of the six algorithms. By directly plotting the classification accuracy curve with the iteration times, we can see that the classification accuracy increases monotonously at each iteration until level off. Figure 2 shows that GCBA converges faster than BBA. From Figure 2, it can be analyzed that GCBA does not solve the shortcoming that BBA falls into the local optimal solution easily. IGCBA falls into the local optimal solution at the 46th iteration, and it jumped out of the local optimal solution at the 66th iteration. Although the ability of IGCBA to jump out of the local optimal solution is not as good as HIDA and HLBDA, it is significantly better than IG-bBOA, BBA and GCBA.

**Figure 3:**
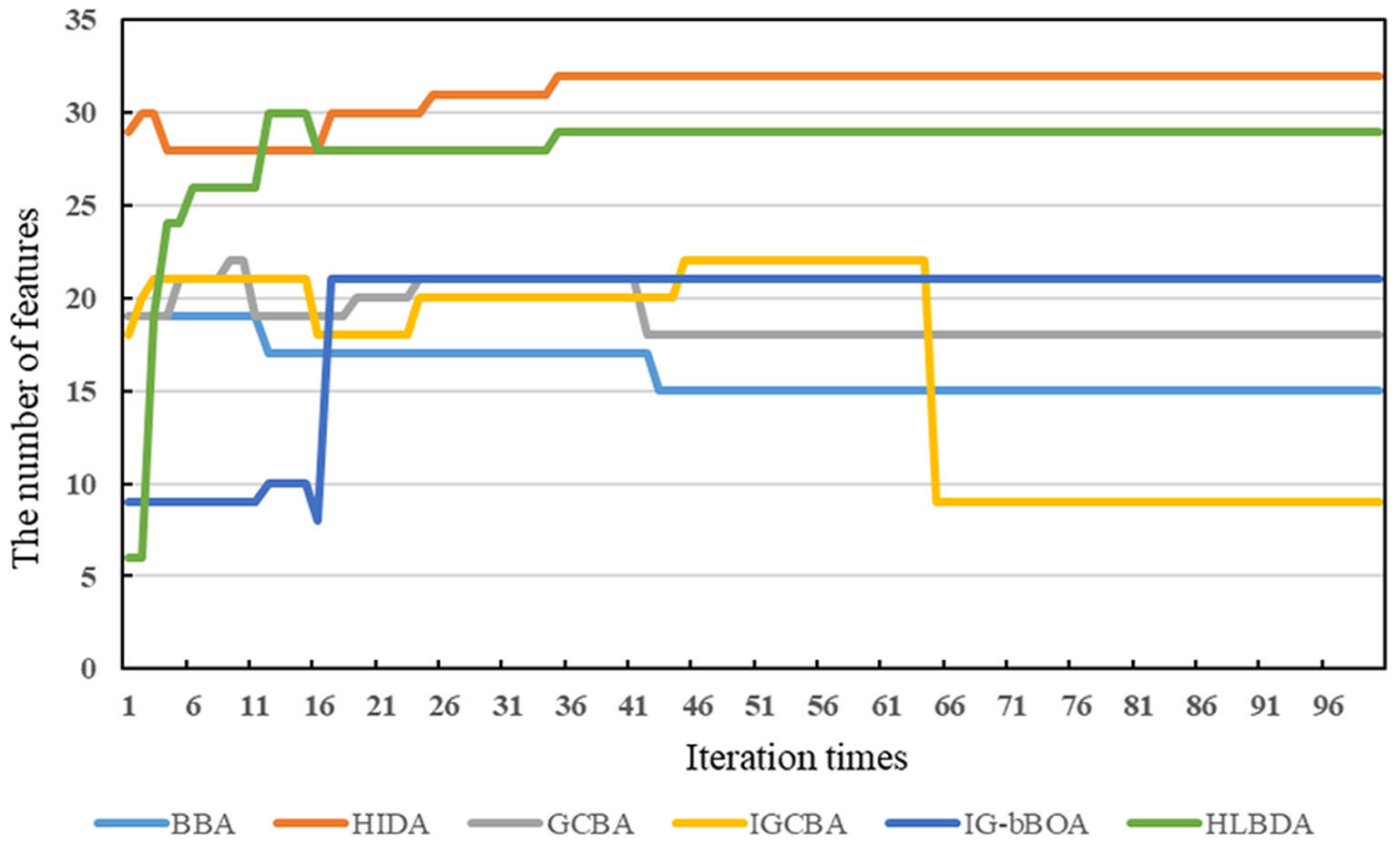
The feature numbers curves of the six algorithms on the survey dataset.

Figure 3 shows that the alteration trend of the number of features selected by the six algorithms in the survey dataset with the number of iterations. Since the non-linear equation balances the exploitation and exploration capabilities of IGCBA, IGCBA has strong exploitation capabilities in the later stage. Therefore, IGCBA finds fewer features at the 65th iterations. Particularly, although the prediction accuracy of HIDA and HLBDA in Figure 2 is 0.12 and 0.12% higher than that of IGCBA, the number of features finally found by IGCBA is 23 and 20 fewer than HIDA and HLBDA. Combining Figures 2, 3, the experimental results show that IGCBA has strong exploitation ability and superior performance in the later optimization stage.

### Analysis of the Degree of Feature Variables on Mental Health

Mean Impact Value (MIV) is currently considered to be one of the best algorithms for evaluating the correlation between input variables and output variables. Sorting the variables according to MIV's absolute value can determine the degree of influence of input variables on network output variables. The symbol of the MIV value represents the relative direction, and the relative importance of the impact is represented by MIV's absolute value.

The IGCBA-BPNN-4 prediction model eliminates irrelevant and redundant features in the original dataset, decreases model running time, and improves the prediction accuracy of the classifier. At the same time, the feature variables that affect mental health are sorted according to the degree of their importance. Table 6 shows that IGCBA-BPBB-4 selects a total of nine feature variables that affect mental health. The nine feature variables are “Have patients with COVID-19 or not in the living place,” “age,” “employment type,” “Have patients with COVID-19 or not in the workplace,” “the work unit is a designated treatment point or not,” “changes in work intensity,” “usual sleep time,” “place of residence,” and “marital status.” We analyze the factors affecting mental health according to their degree of importance.

**Table 6:** Feature variables that affect mental health: sorted by importance.

**Feature**	**MIV value**	**Sorted by importance**
Have patients with COVID-19 or not in the living place	−0.11158	1
Age	0.09567	2
Employment type	0.09161	3
Have patients with COVID-19 or not in the workplace	−0.03947	4
The work unit is a designated treatment point or not	0.03899	5
Changes in work intensity	−0.03493	6
Usual sleep time	−0.02933	7
Place of residence	0.01904	8
Marital status	0.01328	9

Variables in statistics are divided into numerical variables and categorical variables. When considering the impact of input variables on output variables, the direction of the symbol is only meaningful for numerical variables, and has no meaning for categorical variables. Since the variables in this article are mostly categorical variables, the positive or negative influence of the symbol is not considered in this analysis.

In the community transmission stage of the epidemic, according to a study that cluster transmission occurs in multiple communities and families. On average, each patient transmits the infection to 2.2 people (59). When relatives, friends, and nearby people in the living place are determined to be suspected or confirmed cases, people will have psychological problems such as fear and anxiety due to fear of infection.

Patients with COVID-19 are mostly elderly people. Under normal circumstances, the deterioration of body function with age decreases the health levels of the elderly. The elderly are more vulnerable to the threat of diseases because their immune system is relatively weak. In the “Questions and Answers About COVID-19 and the Elderly” on the WHO official website, a clear answer is also given to the question “Who is at risk of severe illness,” that is, the elderly and all ages of people who are diagnosed with diseases such as hypertension, heart disease, lung disease, diabetes or cancer are more likely to suffer from severe illness than others (60).

Differences in employment type lead to differences in the psychological status of medical workers. In contrast with formal medical personnel, temporarily hired medical personnel may show a stronger sense of anxiety and fear during COVID-19. On the one hand, due to the absence of both manpower capital and social capital, temporary medical workers are more likely to engage in low-tech and labor-intensive jobs. The work pressure caused by high labor intensity make easily temporary medical workers prone to anxiety and hostility. On the other hand, most temporary medical workers are exposed to such a severe epidemic for the first time. They lack the work experience and sufficient mental preparation to deal with severe infectious diseases. At the same time, due to the lack of objective cognition of COVID-19, they are in a highly alert state at work, and their anxiety and fear are more prominent.

There are patients with COVID-19 in the workplace, especially the workplace is a designated treatment point for COVID-19, which will have a greater impact on the mental status of medical workers. In face of high-intensity work pressure and the risk of being infected, medical workers are more likely to become a high-risk group with psychological symptoms. Less sleep and poor sleep during COVID-19 can cause sleep disorders, and sleep disorders are often accompanied by symptoms such as depression, tension, anxiety, hostility and irritability (61). For people who do not have a spouse, they cannot get timely help when they encounter difficulties and need a good listener. They are prone to anxiety and depression (62). The farther the place of residence is from the city center, the lower the population density. It is difficult for COVID-19 to spread rapidly in rural areas (63), and people living in rural areas have less fear of COVID-19 than people living in cities.

## Discussion

According to the above observations, we can make a conclusion that the performance of IGCBA-BPNN-4 is better than other algorithms. First, BPNN learns the non-linear relationship between feature variables and prediction results, which improves the accuracy of mental health prediction. The results in Table 5 indicate that the accuracy of the feature selection algorithms combined with BPNN is higher than that of the feature selection algorithms without BPNN, with an average increase of 2.46%. Particularly, the accuracy of IGCBA-BPNN-4 is 4.04% higher than that of IGCBA. Second, the value calculated by MIV is used as the influence weight, which assesses the extent to which feature variables contribute to mental health. It can be seen from Table 6 that through the calculation of MIV, the nine feature variables that affect mental health are sorted by their importance. The top three important factors affecting mental health are “whether there are patients with COVID-19 in the workplace,” “age” and “employment type.” The result corresponds with our expectations. Third, GCBA eliminates irrelevant and redundant features in the original features, which reduces BPNN's complexity. The results in Table 5 indicate that GCBA reduces the number of features in the survey dataset from 32 to 18. Although GCBA selects more features than SR and BBA, it has higher prediction accuracy. Fourth, the non-linear equation in IGCBA balances the exploitation and exploration capabilities of IGCBA, which accelerates the convergence speed of IGCBA and prevents IGCBA from falling into a local optimal solution. It can be seen from Figures 2, 3 that IGCBA does not fall into the local optimal solution due to its certain exploration capabilities in the later stage. As a result, IGCBA obtains a feature subset that can represent as much information as possible of the original features and as few features as possible. The number of features selected by IGCBA is only half of the number of features selected by GCBA. Besides, the prediction accuracy rate of IGCBA is higher than that of GCBA.

It should be pointed out that although many people have been vaccinated against COVID-19, the COVID-19 epidemic is far from over due to the spread of mutant strains. COVID-19 directly endangers people's lives, and it is extremely important to diagnose COVID-19 quickly and accurately. The latest method proposed by Wang et al. (64, 65) may help diagnose COVID-19 more quickly and effectively. In the fight against COVID-19, when the psychological symptoms of medical workers are discovered and intervened in time, the work efficiency of the entire health system will be improved. The algorithm proposed in this article can more effectively predict the mental health of medical staff, and the research results can also be directly used by global public health departments. However, several limitations also exist in our research. First, the data in this article was obtained through an online survey, and this research is an observational study. As a result, self-report problems and recall biases are inevitable to some extent. Secondly, mental health is affected by personal, family, economic, social environment and other factors. The factors affecting mental health in this article are incomprehensive. Finally, some parameters that are set manually are used in our algorithm. The parameters of the neural network are given by experience rather than obtained from adaptive changes or learning. We will solve this problem in future work.

## Conclusions

The accuracy of existing mental health prediction methods is low because the relationship between the feature variables and the prediction results is non-linear and the prediction dataset contains a lot of irrelevant and redundant features. At the same time, current mental health prediction methods cannot estimate the extent to which the feature variables are important to the prediction results. Therefore, this paper proposes IGCBA-BPNN. First, BPNN is introduced to deal with the non-linear problem between prediction results and feature variables, which improves the accuracy of mental health prediction. Second, MIV is introduced to calculate the influence weight, which assesses the extent to which feature variables contribute to mental health. Third, GCBA is introduced to eliminate redundant and irrelevant features in the original features, which reduces the model complexity of BPNN and improves the performance of BPNN. Fourth, a non-linear equation is designed in IGCBA to speeds up the convergence speed of IGCBA and prevents IGCBA from falling into a local optimal solution. Experiment results show that the performance of IGCBA-BPNN is better than existing algorithms. The IGCBA-BPNN prediction model can obtain good results in mental health prediction.

However, IGCBA only reduces BPNN's input dimension. The BPNN's structure is not improved, and the parameters in the BP network is not optimized. Therefore, how to ascertain the number of neural network nodes is an important challenge in the future.

In a word, with the development of swarm intelligence algorithms and neural network technology, the methods based on swarm intelligence algorithms combined with neural networks are playing an increasingly significant role in the field of prediction. In the future health prediction research, the prediction method based on swarm intelligence algorithm combined with neural network will have a wider application prospect.

## Data Availability Statement

The datasets presented in this study can be found in an online repository: https://github.com/Hu-Li/mental-health-dataset.

## Ethics Statement

The studies involving human participants were reviewed and approved by the Ethics Committee of the School of Public Health, Jilin University. The ethics committee waived the requirement of written informed consent for participation.

## Author Contributions

XW and HL came up with the original idea. HL and TW designed this study and provided research methods. CS and XZ completed the data collection and performed the statistical analysis. TW conducted the experiments. XW supervised the research. HL drafted the manuscript. XW, HL, DG, and CD improved the manuscript. All authors contributed to the article and approved the final version.

## Conflict of Interest

The authors declare that the research was conducted in the absence of any commercial or financial relationships that could be construed as a potential conflict of interest.

## Publisher's Note

All claims expressed in this article are solely those of the authors and do not necessarily represent those of their affiliated organizations, or those of the publisher, the editors and the reviewers. Any product that may be evaluated in this article, or claim that may be made by its manufacturer, is not guaranteed or endorsed by the publisher.
