# Supplementary material for: Prediction of Mental Health in Medical Workers During COVID-19 Based on Machine Learning
Source: Front Public Health. 2021 Sep 7;9:697850. doi: 10.3389/fpubh.2021.697850 (PMC8452905; doi:10.3389/fpubh.2021.697850)
Supplement: Supplementary file 1 [file Data_Sheet_1.ZIP › Research data/The description of variables.pdf]

Table S1. The description of variables.

Factors	#	Variable	Description	Values
Demography	1	Gender	Subject's gender	Male = 1<br>Female = 2
	2	Age	Subject's age	Numeric
	3	Place of residence	Subject's place of residence	Region
	4	Town or country	Are you living in a town or a country?	Town = 1<br>Country = 2
	5	Education	Subject's highest level of education	Junior high school = 1<br>Senior high school = 2<br>College = 3<br>University = 4<br>Postgraduate = 5
	6	Marital status	Subject's marital status	Unmarried = 1<br>Married = 2<br>Divorced = 3<br>Widowed = 4
	7	Chronic disease	Are you sunffering from chronic disease?	Yes = 1<br>No = 2
Family	8	The only child	Are you an only child?	Yes = 1<br>No = 2
	9	Have minor children or not	Do you have any minor children?	Yes = 1<br>No = 2
	10	Whether the minor child is an only child	Is your minor child an only child?	Yes = 1, No = 2
	11	Primary caregiver for children	Who is the main caregiver of your children?	Myself = 1<br>Spouse = 2<br>Grandparents = 3<br>Other relatives = 4<br>Others = 5
	12	Primary caregiver for elderly parents	Does someone take care of your elderly parents?	Yes = 1<br>No = 2
	13	Annual family income	Subject's annual family income	Not more than 30,000 = 1<br>30,001-80,000 = 2<br>80,001-12,000 = 3<br>12,001-20,000 = 4<br>20,001-30,000 = 5<br>More than 30,000 = 6
	14	Current job is supported by family	Is your current work supported by your family?	Yes = 1<br>Neither support nor oppose = 2<br>No = 3
Employment	15	Occupation	Subject's occupation	administrative personnel = 1<br>Disease control personnel = 2<br>Doctor = 3<br>Nurse = 4<br>Medical technician = 5<br>Other occupation = 6
	16	Post	Subject's post	front-line personnel = 1<br>Non-frontline personnel = 2

Employment	17	Working years	Subject's working years	Numeric
	18	Type of work unit	What is the type of your work unit?	Disease control institution = 1<br>LEVEL-I medical institution = 2<br>LEVEL-II medical institution = 3<br>LEVEL-III medical institution = 4
	19	Title	Subject's title	Junior = 1<br>Intermediate = 2<br>Associate senior = 3<br>Senior = 4<br>Other = 5
	20	Employment type	Subject's employment type	Temporarily hired = 1<br>Formal = 2
	21	Monthly income	Subject's monthly income	Not more than 2,000 = 1<br>2,001-4,000 = 2<br>4,001-6,000 = 3<br>6,001-8,000 = 4<br>8,001-10,000 = 5<br>10,001-12,000 = 6<br>12,001-15,000 = 7<br>More than 15,000 = 8
	22	Changes in work intensity	Before and after the COVID-19 pandemic, how do you feel about the work intensity?	Greater = 1<br>No change = 2<br>Less = 3
	23	Working hours per week	How many days do you usually work a week during COVID-19?	Not more than 3 days = 1<br>4 days = 2<br>5 days = 3<br>6 days = 4<br>7 days = 5
	24	Satisfaction level with the protective measures	Are you satisfied with the protective measures in the workplace?	Strongly satisfied = 1<br>Satisfied = 2<br>Neutral = 3<br>dissatisfied = 4<br>Strongly dissatisfied = 5
	25	Psychological training	Have you received psychological training during COVID-19?	Often = 1<br>Sometimes = 2<br>Never = 3
Lifestyle	26	Usual sleep time	How long do you usually sleep every day during COVID-19?	Not more than 5 hours = 1<br>About 6 hours = 2<br>About 7 hours = 3<br>About 8 hours = 4
	27	Resting place	Where do you usually rest during COVID-19?	Home = 1<br>workplace = 2<br>Temporary location arranged by the government/work unit = 3
	28	Frequency of exercise	How has your frequency of exercise changed during COVID-19?	Increase = 1<br>No change = 2<br>Decrease = 3

Work/living envi-ronment related to COVID-19	29	Have COVID-19 patients or not in the workplace	Have COVID-19 patients (including suspected patients) ever appeared in your workplace?	Yes = 1<br>No = 2
	30	In close contact with COVID-19 patients in the workplace	Have you been in close contact with COVID-19 patients (including suspected patients) in your workplace?	Yes = 1<br>No = 2
	31	Have COVID-19 patients or not in the living place	Have COVID-19 patients (including suspected patients) ever appeared in your living place?	Yes = 1<br>No = 2
	32	The work unit is a designated treatment point or not	Is your work unit a designated treatment point for COVID-19 patients?	Yes = 1<br>No = 2

The 90 variables named with serial numbers in the dataset correspond to the 90 items in the Self-reporting Inventory (SCL-90). The Self-reporting Inventory is shown in Table S2. Each item of the scale uses a five-level scoring system. The item scale is 5 points, and it represents the level of the question: not at all (one point), a little bit (two points), moderately (three points), quite a bit (four points), and extremely (five points).

Table S2. The Self-reporting Inventory.

Number	Items
1	Headaches
2	Nervousness or shakiness inside
3	Unwanted thoughts or ideas that won't leave your head
4	Faintness or dizziness
5	Loss of sexual interest or pleasure
6	Feeling critical of others
7	The idea that someone else can control your thoughts
8	Feeling others are to blame for most of your troubles
9	Trouble remembering things
10	Worried about sloppiness or carelessness
11	Feeling easily annoyed or irritated
12	Pains in heart or chest
13	Feeling afraid in open spaces or on the street
14	Feeling low in energy or slowed down
15	Thoughts of ending life
16	Hearing voices that other people do not hear
17	Trembling
18	Feeling that most people cannot be trusted
19	Poor appetite
20	Crying easily
21	Feeling shy or uneasy with the opposite sex
22	Feeling of being trapped or caught
23	Suddenly scared for no reason
24	Temper outbursts that you could not control
25	Feeling afraid to go out of your house alone
26	Blaming yourself for things
27	Pains in lower back
28	Feeling blocked in getting things done
29	Feeling lonely
30	Feeling blue
31	Worrying too much about things
32	Feeling no interest in things
33	Feeling fearful
34	Your feelings being easily hurt
35	Other people being aware of your private thoughts
36	Feeling others do not understand you or are unsympathetic
37	Feeling that people are unfriendly
38	Having to do things very slowly
39	Heart pounding or racing
40	Nausea or upset stomach

41	Feeling inferior to others
42	Soreness of your muscles
43	Feeling that you are watched or talked about by others
44	Trouble falling asleep
45	Having to check and double check what you do
46	Difficulty making decisions
47	Feeling afraid to travel on buses, subways or trains
48	Trouble getting your breath
49	Hot or cold spells
50	Having to avoid certain things, places or activities
51	Your mind going blank
52	Numbness or tingling in parts of your body
53	A lump in your throat
54	Feeling hopeless about the future
55	Trouble concentrating
56	Feeling weak in parts of your body
57	Feeling tense or keyed up
58	Heavy feelings in your arms or legs
59	Thoughts of death or dying
60	Overeating
61	Feeling uneasy when people are watching or talking about you
62	Having thoughts that are not your own
63	Having urges to beat, injure or harm someone
64	Awakening in the early morning
65	Having to repeat the same actions such as touching, counting, washing
66	Sleep that is restless or disturbed
67	Having urges to break or smash things
68	Having ideas or beliefs that others do not share
69	Feeling very self-conscious with others
70	Feeling uneasy in crowds such as shopping or at a movie
71	Feeling everything is an effort
72	Spells of terror or panic
73	Feeling uncomfortable about eating or drinking in public
74	Getting into frequent arguments
75	Feeling nervous when you are left alone
76	Others not giving you proper credit for your achievements
77	Feeling lonely even when you are with people
78	Feeling so restless you couldn't sit still
79	Feeling of worthlessness
80	Feeling that familiar things are strange or unreal
81	Shouting or throwing things
82	Feeling afraid you will faint in public
83	Feeling that people will take advantage of you if you let them
84	Having thoughts about sex that bother you a lot
85	The idea that you should be punished for your sins
86	Feeling pushed to get things done
87	The idea that something serious is wrong with your body
88	Never feeling close to another personal
89	Feelings of guilt
90	The idea that something is wrong with your mind
